# *Prg4* and Osteoarthritis: Functions, Regulatory Factors, and Treatment Strategies

**DOI:** 10.3390/biomedicines13030693

**Published:** 2025-03-12

**Authors:** Peng-Jie Fu, Sheng-Yuan Zheng, Yan Luo, Zhuo-Qun Ren, Zi-Han Li, Ya-Ping Wang, Bang-Bao Lu

**Affiliations:** 1 Department of Orthopedics, Xiangya Hospital, Central South University, Changsha 410008, China; 8301210315@csu.edu.cn (P.-J.F.); 8301190423@csu.edu.cn (S.-Y.Z.); 8301190419@csu.edu.cn (Y.L.); 8301200408@csu.edu.cn (Z.-Q.R.); 8301210420@csu.edu.cn (Z.-H.L.); 2 National Clinical Research Center for Geriatric Disorders, Xiangya Hospital, Central South University, Changsha 410008, China; 3 Department of Clinical Medicine, Xiangya Medicine School, Central South University, Changsha 410013, China; 4 Teaching and Research Section of Clinical Nursing, Xiangya Hospital, Central South University, Changsha 410008, China

**Keywords:** Prg4, osteoarthritis, lubricin, TGF-β, transcription factor, Wnt

## Abstract

Proteoglycan 4 (PRG4), also known as lubricin, plays a critical role in maintaining joint homeostasis by reducing friction between articular cartilage surfaces and preventing cartilage degradation. Its deficiency leads to early-onset osteoarthritis (OA), while overexpression can protect against cartilage degeneration. Beyond its lubricating properties, PRG4 exerts anti-inflammatory effects by interacting with Toll-like receptors, modulating inflammatory responses within the joint. The expression of *Prg4* is regulated by various factors, including mechanical stimuli, inflammatory cytokines, transcription factors such as Creb5 and FoxO, and signaling pathways like TGF-β, EGFR, and Wnt/β-catenin. Therapeutic strategies targeting *PRG4* in OA have shown promising results, including recombinant PRG4 protein injections, gene therapies, and small molecules that enhance endogenous *Prg4* expression or mimic its function. Further research into the molecular mechanisms regulating *Prg4* expression will be essential in developing more effective OA treatments. Understanding the interplay between *Prg4* and other signaling pathways could reveal novel therapeutic targets. Additionally, advancements in gene therapy and biomaterials designed to deliver PRG4 in a controlled manner may hold potential for the long-term management of OA, improving patient outcomes and delaying disease progression.

## 1. Introduction

Osteoarthritis (OA) stands as the prevalent type among the various forms of arthritis, exerting an impact on millions of individuals globally. It is a comprehensive joint disease that encompasses all joint tissues. The symptomatic characteristics of OA include the degeneration of articular cartilage, the remodeling of subchondral bone, synovial inflammation, and the formation of osteophytes, as well as the inflammation and fibrosis of the infrapatellar fat pad. These manifestations can lead to symptoms such as pain, joint stiffness, and a decrease in joint function [1]. The pathogenesis of OA is multifactorial, involving age, gender, obesity, joint injuries, and genetic factors. Additionally, joint deformities and mechanical stress also contribute to the development of OA [2]. The proliferation of chondrocytes and their hypertrophic differentiation give rise to the generation of matrix metalloproteinases (MMPs), as well as aggrecanases. These enzymes are responsible for the degradation of the extracellular matrix of cartilage [3]. Recent research has identified potential therapeutic targets for OA. Genetic mouse models have shown that loss-of-function mutations in genes encoding cartilage-degrading enzymes like ADAMTS5 and MMP13 can protect against OA [3,4].

Proteoglycan 4 (PRG4), or lubricin, is a pivotal glycoprotein mainly for joint lubrication and cartilage surface protection. Secreted by synovial fibroblasts and superficial zone chondrocytes, it is crucial in maintaining the articular cartilage’s integrity and function by reducing joint friction [5]. PRG4 is encoded by the *PRG4* gene and is secreted into the synovial fluid, where it forms a lubricating boundary layer on the surface of the articular cartilage [6]. This boundary layer is essential in minimizing wear and tear on cartilage surfaces during joint movement, thereby preserving joint function and preventing degeneration. A deficiency in Prg4 causes early-onset OA, while its overexpression prevents cartilage degeneration in mouse models [7]. Mice lacking Prg4 exhibit significant cartilage degradation and synovial hyperplasia [8].

Conversely, the overexpression of *Prg4* has been shown to prevent cartilage degeneration in experimental models, suggesting its potential therapeutic application [7]. The protective effects of Prg4 are attributed not only to its lubricating properties but also to its ability to inhibit synovial cell proliferation and inflammatory responses within the joint. PRG4 interacts with Toll-like receptors 2 and 4, exerting anti-inflammatory effects and further contributing to its role in human joint health [9]. Therefore, understanding the regulatory mechanisms of *PRG4* expression and its interaction with other molecular pathways in the joint environment could lead to new therapies to preserve joint health and prevent OA progression.

## 2. The Role of Prg4 in Osteoarthritis

The therapeutic potential of Prg4 in OA has been highlighted by experimental models, where the overexpression of *Prg4* or administration of recombinant Prg4 can prevent cartilage degradation [10]. For instance, intra-articular injections of recombinant Prg4 have been shown to restore the lubricating properties of the synovial fluid and protect against cartilage wear in animal models, providing a potential clinical application for Prg4 in OA management [11]. Moreover, genetic studies have identified mutations in the *Prg4* gene that are associated with familial forms of OA, further emphasizing the critical role of *Prg4* in maintaining joint health [12]. In summary, *Prg4* is a vital component in preserving cartilage integrity and joint function, as it provides lubrication and exerts anti-inflammatory effects.

### 2.1. Joint Lubrication

In joints that are in a healthy state, the molecules responsible for lubrication cover the surface of the cartilage. These molecules offer boundary lubrication, and they also stop cells and proteins from sticking to the surface. Arthropathy may occur in individuals who have experienced joint trauma, suffer from inflammatory arthritis, or have a genetically determined lack of lubricin. In such cases, due to the deficiency in lubricin, the articular cartilage is subjected to damage [13].

**Figure 1:**
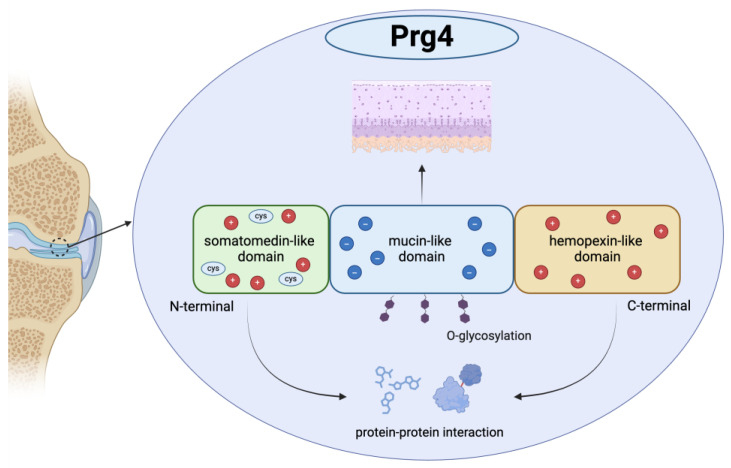
A schematic shows the Proteoglycan 4 (Prg4) structure. Prg4 has important functional and structural domains: a central mucin-like domain flanked by an N-terminal cysteine-rich somatomedin-like domain and a C-terminal hemopexin-like domain. Its central domain is negatively charged, with positively charged termini. The N- and/or C-terminus helps Prg4 to attach to joint surfaces via interactions with other macromolecules. Protein–protein interactions occur due to the mucin domain’s folding. Glycosylated structures form a boundary layer, generating repulsion and an anti-adhesion effect.

Prg4 comprises distinct domains that are crucial for its function and structure. It features a central mucin-like domain, which is flanked by a cysteine-rich N-terminal somatomedin-like domain and a C-terminal hemopexin-like domain [14]. The N-terminus, C-terminus, or both facilitate their attachment to the joint surface through interactions with other macromolecules [15], leading to protein–protein interactions with Prg4, which folds with the mucin domain, forming a boundary layer [14]. The lubricating properties of Prg4 are primarily due to its ability to form a boundary layer on the surface of the articular cartilage [16]. Moreover, lubricin harbors a core 1-oxo-glycan architecture along with sialic acid residues. These components endow the protein with a centrally located negatively charged domain, which is flanked by positively charged terminal regions [17] (Figure 1). This glycan component may contribute to the protein’s low friction and non-adhesive lubrication properties [18]. Glycosylation plays an important role in regulating the biological effects of Prg4. It is structured to form a nanolayer that creates repulsive forces and imparts an anti-adhesion effect [19]. Patients with arthritis have increased PRG4 sialylation, which may lead to altered PRG4 glycosylation [17].

Studies have shown that mice lacking Prg4 develop early-onset OA, characterized by heightened joint friction, cartilage erosion, and increased susceptibility to mechanical damage [7]. Furthermore, chondrocytes in the superficial zone just below the cartilage surface have limited replicative capacity [20], and, in the case of insufficient lubricin, enhanced mechanical strains from increased friction may be felt [21]. Friction stimulates the activation of caspase-3, leading to arthropathy [22].

Loss-of-function alterations in the *PRG4* gene are the causative factors for the development of camptodactyly-arthropathy-coxa vara-pericarditis (CACP) syndrome [12]. People with CACP syndrome experience premature joint failure [12], and the synovial fluid in CACP patients is unable to reduce the friction between synthetic and cartilage bearings [23]. Studies have shown that friction and chondrocyte apoptosis are directly related, and both are reduced in the presence of lubricin [24]. *Prg4* KO mice had joint surface cell morphology changes at 2 weeks old and progressive joint surface destruction at 16 weeks old [8]. In addition, apoptosis-related markers were increased in the knee joint tissue of *Prg4* KO mice [23]. This reveals the significance of lubricin in providing boundary lubrication, which plays a crucial role in preserving the structural and cellular integrity of the cartilage surface. A number of research works have demonstrated that the intra-articular injection of either natural lubricin or lubricin expressed through recombinant technology can exert disease-altering impacts in rodent models that simulate post-traumatic OA [25,26,27,28].

In summary, Prg4 is essential for joint homeostasis by maintaining chondrocyte integrity and reducing friction. The further understanding of lubricin and its molecular mechanisms will help us to develop more effective strategies to treat joint diseases such as OA and maintain joint health.

### 2.2. Anti-Inflammatory Effects

**Figure 2:**
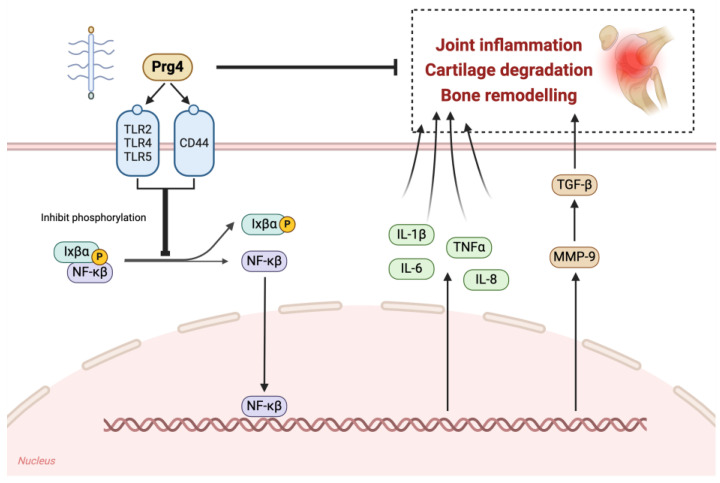
Schematic diagram of the molecular pathways by which Prg4 inhibits joint inflammation, cartilage degeneration, and bone remodeling. Prg4 exerts anti-inflammatory effects by binding to TLR2, -4, and -5 on the surfaces of synoviocytes and can also be mediated through the CD44 receptor. After PRG4-CD44 interaction, it inhibits IκBα phosphorylation after IL-1 receptor stimulation, causing the IκBα subunit to remain in the cytoplasm, inhibiting NF-κB nuclear translocation in human synovial fibroblasts, thereby inhibiting the NF-κB-MMP9-TGF-β pathway, as well as the expression of IL-6, IL-8, IL-1β, TNF-α, and other inflammatory factors.

Prg4 plays a critical role not only in maintaining cartilage integrity but also in modulating inflammatory responses within the joint. The anti-inflammatory effects of Prg4 are mediated through its interactions with key inflammatory pathways. PRG4 can bind to Toll-like receptors (TLRs) 2, 4, and 5 on the surfaces of human synovial cells [9,29]. In experimental models, the anti-inflammatory properties of Prg4 have been demonstrated; its overexpression could downregulate the expression of inflammatory factors (IL-1β and IL-6) [30]. The anti-inflammatory effect of PRG4 is mediated through the CD44 receptor, because blocking this receptor can block the effects of rhPRG4 on matrix metalloproteinase-9, matrix metalloproteinase-13, ribanase-2, IL-6, IL-8, and COX2 expression [31]. The interaction between PRG4 and CD44 restrains the nuclear translocation of nuclear factor kappa B (NF-κB) within human synovial fibroblasts. Moreover, it lessens the IL-1β-induced proliferation of synoviocytes and the expression of matrix-degrading enzymes [31]. Prg4 inhibits IκBα phosphorylation following IL-1 receptor stimulation. IκBα binds to the NF-κB subunit, causing its cytoplasmic retention [32]. Yuji Maenohara et al. found that lubricants maintain SFZ by inhibiting MMP9 expression, and the NF-κB-MMP9-TGF-β signaling pathway is believed to potentially affect *Prg4* expression. In the articular cartilage, MMP9 activates TGF-β signaling; it can promote the release of TGF-β in murine articular cartilage from a complex composed of active TGF-β ligands, latency-related peptides, and latent TGF-β-binding proteins, thereby activating TGF-β and affecting downstream cell activity [33,34] (Figure 2). Studies have indicated that Prg4 can mitigate the destruction of articular cartilage and the apoptosis of chondrocytes. Additionally, it can promote the regeneration of articular cartilage in models of inflammatory arthritis [35]. In the synovia of *Prg4*^−/−^ mice, the expression level of pro-inflammatory macrophages was notably higher compared to that of anti-inflammatory macrophages [36]. Restoring the expression of *Prg4* leads to a shift in macrophages towards an anti-inflammatory phenotype. This shift not only decreases the occurrence of synovitis but also mitigates synovial fibrosis in mouse models [37]. In summary, these studies support an immunomodulatory role for Prg4.

Synovitis represents a prevalent characteristic during the onset and progression of OA. The activation of innate immune cells and the complement system serves as the primary factor propelling the inflammation of synovial tissue and the degeneration of cartilage in the context of OA [38]. Alkuleni et al. showed that the amounts of p50 and p65 were greater in *Prg4*^−/−^ synoviocytes as compared to those in *Prg4*^+/+^ synoviocytes. This finding is in line with the observation that the expression levels of CD44 were higher in *Prg4*^−/−^ mice’s synoviocytes [31], expanding the role of PRG4-CD44 in OA pathogenesis.

## 3. Regulation of *Prg4* Expression

The regulation of *Prg4* expression is complex, involving mechanical stimuli, inflammatory cytokines, transcription factors, and growth factors (Table 1). Multiple studies have demonstrated that these factors affect the expression of *Prg4* through different pathways (Table 2). Cyclic mechanical loading increases *Prg4* expression in bovine articular chondrocytes. Conversely, mechanical unloading or joint immobilization leads to decreased *Prg4* expression, contributing to cartilage degradation and OA progression [39]. Inflammatory cytokines, particularly interleukin-1β (IL-1β) and tumor necrosis factor-alpha (TNF-α), also regulate *Prg4* expression. Elevated levels of these cytokines in OA downregulate *Prg4* expression in synovial fibroblasts and chondrocytes, exacerbating inflammation and cartilage degeneration [26]. Transcription factors such as Sox9 are essential for chondrogenesis and cartilage homeostasis and directly enhance *Prg4* transcription by binding to its promoter [40]. Growth factors like transforming growth factor-beta (TGF-β) promote *Prg4* expression in chondrocytes and synovial cells, maintaining joint lubrication and integrity [41]. There is no evidence that there is a direct link between the epigenetic modification of DNA methylation and *PRG4*. However, 5-aza (a DNA methyltransferase inhibitor) may indirectly affect the expression of *PRG4* in gingival fibroblasts by interfering with the expression of TGF-βRII in the TGF-β signaling pathway [42]. Understanding the molecular regulatory mechanisms of *Prg4* may provide insights into the pathophysiology of joint diseases and highlight potential therapeutic targets to enhance *Prg4* expression to maintain joint health.

### 3.1. Transforming Growth Factor (TGF)-β Signaling

**Figure 3:**
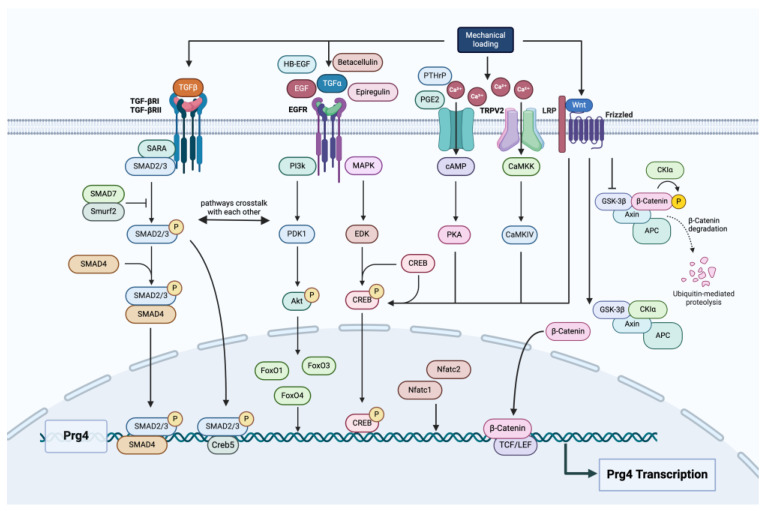
Schematic diagram representing the molecular pathways that regulate *Prg4* expression.

TGF-β exerts its effects on *Prg4* expression through the activation of the SMAD signaling pathway. Upon TGF-β binding to its receptors (TGF-βRI and TGF-βRII) on the cell surface, the receptors phosphorylate SMAD2 and SMAD3. These phosphorylated SMADs combine with SMAD4 to form a complex. Subsequently, this complex migrates into the nucleus, where it modulates the transcription of target genes, among which is *Prg4* [51]. In addition to the SMAD pathway, TGF-β signaling can also activate non-SMAD pathways, such as the MAPK, PI3K/Akt, PKA-CREB, and RhoA/ROCK pathways [52]. These pathways interact with the SMAD signaling cascade to fine tune the cellular responses to TGF-β. TGF-β and SMAD3 also regulate the alternative splicing of *Prg4*, thereby changing the structure of the Prg4 molecule and affecting its degree of binding to the extracellular matrix [53] (Figure 3). Moreover, the mechanical loading of cartilage increases the sensitivity of chondrocytes to TGF-β, enhancing *Prg4* expression [54]. When there was dominant-negative interference with the TGF-β signaling pathway in mouse cartilage, it led to a reduction in the expression of both OA-related mRNA and *Prg4* mRNA. This outcome implies that *Prg4* might play a role in mediating certain aspects of the protective effects that TGF-β exerts on cartilage tissue [55]. Autologous platelet-rich fibrin activates TGF-β signaling, phosphorylates Smad3, and upregulates *PRG4* in human fibroblasts. It promotes articular cartilage repair and serves as an effective treatment for OA [56].

### 3.2. Wnt Signaling

The Wnt signaling pathway is among the most extensively investigated in the fields of developmental biology and the pathophysiology of numerous diseases. Genes belonging to the Wnt family code for a group of roughly 20 small secreted proteins. These proteins exhibit a high degree of conservation across different organisms [57]. The Wnt receptor is a low-density lipoprotein-related receptor 5 (LRP5) that was shown to be involved in the regulation of bone mass. Wnt signaling has received widespread attention, and its functions have been extensively studied [58]. The Wnt signaling pathway encompasses two principal subtypes: the canonical pathway and the non-canonical pathway. Particular ligands, among which Wnt1 and Wnt3a are notable examples, engage in interaction with a complex that is constituted by the seven-transmembrane receptor Frizzled (FZD) and the single-transmembrane coreceptor LRP5/6. This molecular interaction event instigates the activation of the canonical Wnt signaling cascade, thereby triggering a series of downstream cellular responses [59]. The non-canonical signaling pathway is a collective term referring to those pathways that are not mediated by β-catenin. Ligands like Wnt5a and Wnt11 can activate the Wnt/Ca^2+^ and Wnt/planar cell polarity (PCP) pathways. Significantly, this activation occurs without causing the accumulation of intracellular β-catenin [57].

Studies have shown that Wnt7b expression is most closely linked to OA and RA. It is elevated in OA articular cartilage and RA synovia, offering potential therapeutic targets for these common joint diseases [60]. Moreover, an increase in the expression level of Wnt 1-induced signaling pathway protein 1 (WISP-1) was observed not only in the cartilage and synovia of human patients with OA but also in the cartilage of mice with OA [61]. The loss of β-catenin function in cartilage induces osteoarthritic changes in mice. It significantly increases the number of apoptotic cells [62] and causes the loss of the superficial zone along with a decrease in *Prg4* expression, highlighting β-catenin’s key role in cartilage integrity and osteoarthritis pathogenesis [63]. Wnt3a stimulates the activation of β-catenin and increases the expression of MMP3, MMP13, ADAMTS4, and ADAMTS5, leading to cartilage matrix degradation [64]. In addition, mice with the complete knockout of Wnt16, a gene that shows notable upregulation in the articular cartilage of both OA patients and mouse models of OA, exhibited the more pronounced degradation of the articular cartilage compared to wild-type mice following the destabilization of the medial meniscus (DMM). In these knockout mice, there was an increase in chondrocyte apoptosis, and the expression level of *Prg4* was reduced [65].

Xu et al. recently found that, in conditional β-catenin KO mice, *Prg4* expression in superficial zone (SFZ) cells was reduced, while β-catenin stably induced *Prg4* expression [44]. The mRNA expression of Wnt5a, Wnt5b, and Wnt9a is induced by mechanical stress loading. The Wnt/β-catenin-induced enhancement of CREB transcription and PKA pathway-induced CREB phosphorylation may both be involved in *Prg4* induction by mechanical loading in mouse models [66] (Figure 3).

### 3.3. EGFR Signaling

Epidermal growth factor receptor (EGFR) is an extracellular protein ligand and a transmembrane receptor that is a member of the epidermal growth factor (EGF) family [67]. EGFR can be bound and activated by a diverse range of ligands. These ligands encompass epidermal growth factor (EGF), transforming growth factor-α (TGFα), amphiregulin, heparin-binding EGF (HB-EGF), epiregulin, and betacellulin [68]. The signaling pathway of EGFR is of great significance in the processes of endochondral ossification, the differentiation of chondrocytes, and the formation of the growth plate [69]. Moreover, multiple studies have shown that the EGFR signaling pathway is intricately involved in regulating *Prg4* expression in articular cartilage tissue. This likely plays a protective role in shielding joints from damage, helping to maintain joint integrity and function [70,71]. Some studies have demonstrated that TGF-β1 and bone morphogenetic protein 7 (BMP-7) serve as efficient inducers of *Prg4* in synovial explants derived from calf synovial tissue [72]. Jia et al. found that, in cartilage-specific EGFR-deficient mouse joints, *Prg4* signaling was nearly absent on relevant surfaces, likely due to the direct regulatory effect of EGFR signaling on *Prg4*, indicating its importance in cartilage tissue’s *Prg4* expression and function [45].

Mitogen-inducible gene 6 (*Mig-6*), alternatively referred to as gene 33 or ErbB receptor feedback inhibitor 1 (ERRFI1), is located within the cytoplasm [73]. The *Mig-6* protein engages in interactions with the EGFR signaling pathway and exerts a suppressive effect on it through a two-tiered mechanism. Firstly, it hinders the catalytic function of EGFR. Secondly, it leads to a decrease in the concentration of the EGFR receptor [74]. Studies of the chondrocyte-specific knockout of *Mig-6* support that EGFR signaling is required to obtain enough superficial chondrocytes by retaining their proliferative capacity, and Prg4 is reduced in the articular cartilage of *Mig-6*-overexpressing mice [75]. However, the specific downstream EGFR signaling molecules inducing *Prg4* expression remain unclear. Thus, more thorough and in-depth exploration is required in future research to clarify the underlying mechanisms and potentially identify new regulatory targets.

### 3.4. Tryptase β

Tryptase β belongs to serine proteases and represents the secretory granule protein with the highest abundance within human mast cells (MCs) [76]. MCs assume a pivotal function in modulating the host’s responses and maintaining tissue homeostasis. They achieve this by secreting powerful signaling molecules like histamine and tryptase β. In recent research, these molecules have been discovered to have a connection with osteoarthritis [77]. When mast cells are depleted or their activity is blocked, the progression of OA is delayed [78]. A study analyzing more than 8500 OA knee samples found an association between antihistamine use and reduced OA severity in the patient population [79]. Das et al. found that this was similar to the effects observed in preclinical models of OA treated with PRG4; their study revealed that tryptase β cleaved PRG4 at multiple sites in human synovial fluid, causing the loss of its lubrication potential. PRG4 can activate NF-κB via TLR2, TLR4, and TLR5, and the cleaved PRG4 further intensifies NF-κB activation in human primary synovial fibroblasts [49]. Following a joint injury, pro-inflammatory substances like TNF-α and IL-1β exert a direct effect on mast cells. This direct action leads to an elevation in the expression and subsequent secretion of tryptase β by these mast cells [80]. Tryptase β weakens lubrication activity by cleaving PRG4, associated with increased synovitis and cartilage damage. Allosteric anti-tryptase antibodies with a high degree of selectivity have been developed, and these antibodies are capable of suppressing the activity of human tryptase β [81]. In OA-related inflammatory states, or when the PRG4 levels are low, intra-articular treatment with trypsin inhibitors and PRG4 may help to keep the cartilage healthy and possibly slow OA progression [49].

### 3.5. FoxO

FoxO proteins constitute a family of transcription factors that have been conserved throughout evolution and possess crucial roles in processes such as development, the aging process, and determining longevity. In the context of mammals, the FoxO family is composed of four members (FoxO1, FoxO3, FoxO4, and FoxO6). These members exhibit both unique functions and some overlapping functions among them [82]. Moreover, the transcription factors of the FoxO family act as the downstream targets for the protein kinase Akt. The PI3K-Akt-FoxO signaling cascade is involved in diverse cellular functions. These functions encompass cell proliferation and the maintenance of cell survival [83] (Figure 3). Meniscal damage is widely recognized as a significant contributor to the progression of OA. Among the factors involved, FoxO1 and FoxO3 are known to play crucial roles in the development of the meniscus and the maintenance of its homeostasis, especially during the processes of aging and in the context of OA [84]. A research effort that involved the analysis of Col2Cre-FoxO1, -3, and -4 single-KO mice, as well as triple knockout mice (Col2Cre-TKO), revealed that both Col2Cre-TKO mice and AcanCreERT-TKO mice exhibited a decreased cell density in the surface layer of the knee joint cartilage. Moreover, the level of Prg4 in these mice was notably reduced. In an in vitro setting, the ectopic expression of FoxO1 was found to trigger the activation of *Prg4* expression, and FoxO1 was observed to act synergistically with TGF-β [46]. These findings confirm the important role of FoxO family members in joint homeostasis and the prevention of osteoarthritis.

### 3.6. Creb5

Shear stress generated by fluid flow can specifically trigger the expression of *Prg4* in explants obtained from the superficial area of bovine articular cartilage. This tightly confined induction phenomenon indicates that the superficial cells may have a unique transcription factor responsible for the expression of *Prg4*. Research using mouse findings has demonstrated that mechanical motion leads to the phosphorylation of the DNA-binding transcriptional regulator Creb, which in turn promotes an increase in *Prg4* expression [66]. Creb5 is a specialized transcription factor that is expressed in the superficial zone articular cartilage of both cattle and human beings. It plays a crucial role in activating the expression of *Prg4* when responding to the TGF-β and EGFR signaling pathways. Among transcription factors, the Creb5 gene stands out as the most distinct transcriptional regulator. The levels of Creb5 in superficial-zone chondrocytes are roughly 25 times higher compared to those in deep-zone chondrocytes [85]. Furthermore, the cited study found that both the TGF-β and EGFR signaling pathways could enhance the function of Creb5. Specifically, they can promote the expression of *Prg4* in chondrocytes. In newborn bovine knee joint superficial chondrocytes, the EGFR and TGF-β signaling pathways contributed to an increase in the phosphorylation level of Creb5. Additionally, TGF-β significantly enhances the binding of Creb5 to the promoter-proximal regulatory elements (E1 and E2) of *Prg4* [85] (Figure 3). In subsequent studies, by comparing the expression of Wnt5a, Wnt4, and Wnt11 in Creb5 knockout and overexpression mice, it was demonstrated that Creb5 can not only maintain the expression of *Prg4* but also inhibit the positive and negative feedback loops of Wnt5a in the perichondrium, hindering cartilage hypertrophy and osteogenesis [47]. The results from in vitro experiments suggest that Creb5 is of great significance in the regulation of *Prg4* expression. As a result, the correlation between Creb5 and the expression of *Prg4* requires in vivo examination.

### 3.7. Nuclear Factor of Activated T Cells (NFATc)

Multiple studies have delved into the pivotal function of the nuclear factor of activated T cells (NFATc) transcription factor in articular cartilage and the regulation of *Prg4* expression. These investigations have demonstrated that Nfatc1 exhibits robust expression in the surface area of murine articular cartilage [86]. Nfatc1 plays a vital role in maintaining the metabolic balance of articular chondrocytes. In young adult mice, the expression level of Nfatc1 is relatively high. It can effectively regulate the balance between anabolic and catabolic genes, enabling articular chondrocytes to remain in a normal metabolic state. In joint-specific conditional Nfatc1 KO mice, the chondrocytes remain unaffected. In contrast, in Nfatc2 KO mice, symptoms resembling those of OA manifest. This is attributed to a disruption in the equilibrium between the degradation and synthesis of the cartilage matrix [87]. Mice that have undergone a cartilage-specific deletion of Nfatc1 and have a deficiency in Nfatc2 show a decrease in *Prg4* expression within the joint tissue. These mice are also characterized by the development of spontaneous and severe OA, with this condition occurring in 100% of the affected individuals [88].

### 3.8. Mechanical Loading

Moderate physiological mechanical loading, such as that experienced during normal joint movement, positively regulates *Prg4* expression. Studies have demonstrated that the dynamic loading of cartilage stimulates Prg4 secretion by calf chondrocytes, particularly in the superficial zone, where lubricin is predominantly synthesized [11,89]. This is supported by in vitro experiments showing that the mechanical compression of bovine articular cartilage explants upregulates *Prg4* mRNA expression and protein secretion, helping to maintain low-friction joint articulation and protecting the cartilage from mechanical damage [90]. In contrast, excessive mechanical loading—such as that observed in traumatic joint injuries or abnormal joint alignment—has been associated with a reduction in *Prg4* expression. Studies utilizing animal models of post-traumatic osteoarthritis have demonstrated the downregulation of *Prg4* following joint injury, which results in increased cartilage friction and accelerated degeneration [26]. High-impact loading can inhibit Prg4 synthesis by chondrocytes, thereby disrupting the boundary lubrication system and promoting cartilage wear and inflammation. For instance, in mouse models subjected to excessive mechanical loading, cartilage erosion and joint inflammation were observed, accompanied by a significant reduction in *Prg4* expression [8].

The regulation of *Prg4* expression in response to mechanical loading is mediated through complex mechanotransduction pathways. Mechanical signals are converted into biochemical responses primarily through integrins, ion channels, and growth factor signaling pathways, such as the TGF-β pathway. In human chondrocytes, TGF-β has been shown to be activated by mechanical loading, and it plays a critical role in maintaining *PRG4* expression in chondrocytes [48]. Prostaglandin E2 (PGE2) and parathyroid hormone-related peptide (PTHrP) play roles in the induction of *Prg4* by mechanical loading in mouse models [66]. The transient receptor potential vanilloid 2 (TRPV2) channel is one of the mechanoreceptors involved in the induction of *Prg4*. TRPVs belong to the calcium ion channels, and TPRV2 is expressed in the superficial and middle layers of the normal articular cartilage in both humans and mice. However, as aging occurs or OA progresses, the expression level of TPRV2 decreases. In vitro experiments have shown that calmodulin-dependent protein kinase kinase (CaMKK) and calmodulin-dependent protein kinase IV (CaMKIV) are responsible for mediating the induction of *Prg4*. This mediation is achieved through CREB, which is located downstream of the TRPV2 pathway [91]. Furthermore, mechanical loading can influence the expression of *Prg4* via the Wnt/β-catenin signaling pathway. Studies have shown that, in mouse chondrocytes, shear stress loading leads to an increase in the mRNA levels of Wnt5a, Wnt5b, and Wnt9a. Moreover, both recombinant Wnt5a and Wnt5b have been found to enhance the expression of *Prg4* [44] (Figure 3).

## 4. OA Therapies Based on *Prg4*

At present, *PRG4* has been confirmed as a possible therapeutic target in a variety of diseases, and it also has a certain degree of therapeutic potential in OA. *Prg4*^−/−^ mice exhibit cartilage degeneration, resulting in defects in bone morphology [92]. The intra-articular injection of PRG4 derived from human synovial cells in culture reportedly inhibited cartilage degeneration and pain-related behaviors in an anterior cruciate ligament rat model [28], but multiple injections are required to maintain efficacy as the protein is cleared from the synovial fluid [93]. Multiple research studies have shown that when Prg4 is injected into the injured joints of mice [92], rats [26], and pigs [94], there is a notable reduction in the advancement of OA. This treatment approach directly replenishes the insufficient or malfunctioning Prg4 within the joint. By doing so, it offers prompt protective effects against mechanical stress, thereby alleviating the negative impacts on the joint caused by such stress. Moreover, there is evidence indicating that the application of combined gene therapy with IL-1Ra and *Prg4* can provide extended physiological and functional protection in post-traumatic OA models. Additionally, compared to using either IL-1Ra or *Prg4* alone in gene therapy, the co-delivery of a helper-dependent adenovirus (HDV) that expresses both IL-1Ra and *Prg4* demonstrated the more effective preservation of the articular cartilage in a mouse model of posttraumatic OA [10]. Previous research has confirmed the inverse relationship between IL-1 and Prg4 expression. Higher IL-1 leads to lower *Prg4*, and vice versa. In a mouse ACLT model, IL-1Ra treatment boosted *Prg4* expression. In a Yucatan minipig DMM model, rhPRG4 treatment decreased IL-1β in the serum and synovial fluid. These results show the interplay between IL-1 and *Prg4* as potential therapeutic targets for joint conditions [93]. It has been documented that exogenous recombinant human (rh) PRG4E can elevate the expression of VEGF, enhancing blood flow via a mechanism that depends on Toll-like receptors. As a result, this process facilitates the closure of ear wounds and promotes tissue regeneration in mice [95]. The full-length rhPRG4 synthesized by CHO-M cells and the nhPRG4 isolated from OA patients’ fibroblast-like synoviocyte culture supernatants have been experimentally shown to interact with TLR2 and TLR4. This interaction enables them to mediate anti-inflammatory factor activity [9].

Small-molecule modulators that enhance endogenous *Prg4* expression or mimic its function are also under investigation. These compounds aim to upregulate *Prg4* synthesis in chondrocytes and synovial cells or bind to cartilage surfaces, providing long-term joint protection. Recent studies have proven that the anti-inflammatory drug 5-aminosalicylic acid can downregulate the expression of interleukin-6 and cyclooxygenase-2 while upregulating *PRG4* gene expression, which has therapeutic potential in human inflammatory osteoarthritis models [96]. A novel drug-like small molecule called Kartogenin (KGN) has been discovered to significantly boost the expression of *Prg4* in murine superficial joint cells. Due to this property, KGN emerges as a highly potent agent for the treatment of OA. Moreover, it holds potential for application in strategies related to limb regeneration and tissue repair [97]. Highly selective allosteric anti-tryptase antibodies have been developed, and these antibodies are capable of inhibiting the activity of human tryptase β. Combining tryptase β inhibitors with intra-articular PRG4 injection is a potential OA treatment strategy [49,81]. The intra-articular injection of BMP-7 has the effect of suppressing the advancement of OA in rabbits that have undergone ACL resection [98]. Moreover, a phase 1 clinical study focusing on the use of BMP-7 in patients with OA has reported that the safety and tolerability of BMP-7 were within acceptable ranges [99]. Fibroblast growth factor (FGF)-18 is another chondroprotective cytokine. It is expressed in the SFZ and has the ability to upregulate the expression level of *Prg4* in calf chondrocytes [100]. Injecting recombinant FGF-18 into the articular cavity can prevent the progression of OA in rat models [100]. Additionally, tissue engineering approaches that combine Prg4 with biomaterials are being explored. Hydrogels and scaffolds impregnated with Prg4 or *Prg4*-expressing cells can be implanted into damaged joints, providing sustained lubrication and facilitating cartilage repair. These advanced biomaterials mimic the native joint environment, promoting the integration and function of the implanted cells [101,102].

In conclusion, therapies targeting *PRG4* provide innovative solutions for OA treatment by enhancing joint lubrication and protecting the cartilage. Gene therapy, recombinant protein delivery, small-molecule modulators, and tissue engineering approaches hold significant promise in alleviating OA symptoms and slowing disease progression. Continued research and clinical trials are critical to translate these preclinical findings into effective OA treatments.

## 5. Conclusions and Perspectives

Previous studies have achieved significant progress in elucidating the molecular mechanisms underlying cartilage diseases, including OA and RA. Nevertheless, the discovery of genuinely effective treatments for OA remains a formidable challenge, and the complete curing of patients with this condition is still an elusive goal. PRG4, also known as lubricin, is a crucial component in maintaining joint homeostasis. Its role in OA has drawn extensive attention, and numerous investigations have uncovered its mechanistic significance and its potential as a therapeutic target. The TGF-β signaling pathway plays a pivotal role in the regulation of *Prg4* expression. The transcription of *Prg4* is modulated via both the SMAD and non-SMAD pathways, which work together to ensure that appropriate levels of Prg4 are present in the joints [51]. Mechanical loading and many other signaling pathways also contribute to the regulation of *Prg4* [43,44,45,47,48]. The expression of *Prg4* in joint tissue is pivotal in maintaining the homeostasis of articular cartilage. Elucidating the molecular mechanisms that regulate *Prg4* expression could profoundly enhance our comprehension of the physiological and pathological states of articular chondrocytes. Additionally, identifying the transcription factors that govern *Prg4* expression would offer valuable insights into the molecular pathogenesis of OA, potentially paving the way for the development of targeted etiological treatments.

## Figures and Tables

**Table 1:** Regulation factors of *Prg4* expression.

	*Prg4*
Upregulation factor	Transforming growth factor (TGF)-β signaling
	Wnt signaling
	EGFR signaling
	FoxO transcription factor
	Creb 5
	Nuclear factor of activated T cells (NFATc)
	Mechanical loading
Downregulation factor	Tryptase β

**Table 2:** Regulation factors and regulation pathways.

Regulation Factor	In Vitro/In Vivo	Model	Regulation Pathway	Reference
IL-1-alpha, IGF-I, and TGF-β1	In vitro	Bovine articular cartilage explants	IL-1α inhibited *Prg4* expression in chondrocytes, while TGF-β1 stimulated it	[43]
Wnt/β-catenin signaling	In vivo	TOPGAL mice	Mechanical loading and Wnt/β-catenin activation raised Creb1 mRNA levels	[44]
EGFR signaling	In vitro	Cartilage-specific EGFR-deficient mouse; superficial chondrocyte and cartilage explant	Promotes chondrogenic *Prg4* expression and stimulates cartilage surface lubrication	[45]
FoxO	Both in vitro and in vivo	Col2Cre-FoxO1, -3, and -4 single-knockout and triple-knockout mice; immature mouse articular chondrocytes and ATDC5 cells	Activates *Prg4* expression and synergizes with TGF-β	[46]
Creb 5	In vitro	Bovine articular chondrocytes	Promotes *Prg4* expression via TGF-β and EGFR signaling; blocks Wnt5a positive feedback loop in perichondrium	[47]
Mechanical loading	In vitro	Chondrocytes isolated from human femoral heads	Enhancing TGF-β1 action upregulates *PRG4* expression	[48]
Tryptase β	In vivo	Destabilization of the medial meniscus model of OA in rats	Tryptase β modulates joint lubrication in OA by cleaving Prg4	[49]
cAMP	In vitro	TGF-β-stimulated human OA synoviocytes	Increasing intracellular cAMP led to elevated *PRG4* expression and production by OA synoviocytes under TGF-β1 stimulation	[50]

## Data Availability

Not applicable.

## Abbreviations

The following abbreviations are used in this manuscript:

OA	Osteoarthritis
MMPs	Matrix metalloproteinases
Prg4	Proteoglycan 4
CACP	Camptodactyly-arthropathy-coxa vara-pericarditis
NF-κB	Nuclear factor kappa B
IL-1β	Interleukin-1β
TNF-α	Tumor necrosis factor-alpha
TGF-β	Transforming growth factor-beta
LRP5	Lipoprotein-related receptor 5
FZD	Frizzled
WISP-1	Wnt 1-induced signaling pathway protein 1
DMM	Destabilization of the medial meniscus
SFZ	Superficial zone
EGFR	Epidermal growth factor receptor
BMP-7	Bone morphogenetic protein-7
Mig-6	Mitogen-inducible gene 6
ERRFI1	ErbB receptor feedback inhibitor 1
MCs	Mast cells
NFATc	Nuclear factor of activated T cells
PGE2	Prostaglandin E2
PTHrP	Parathyroid hormone-related peptide
TRPV2	Transient receptor potential vanilloid channel 2
CaMKK	Calmodulin-dependent protein kinase kinase
KGN	Kartogenin
FGF	Fibroblast growth factor
